# Draft Genome Sequence of *Pseudomonas aeruginosa* Strain N002, Isolated from Crude Oil-Contaminated Soil from Geleky, Assam, India

**DOI:** 10.1128/genomeA.00104-12

**Published:** 2013-02-07

**Authors:** Abhjit Sarma Roy, Reshita Baruah, Dhrubajyoti Gogoi, Maina Borah, Anil Kumar Singh, Hari Prasanna Deka Boruah

**Affiliations:** Department of Biotechnology, Council of Scientific and Industrial Research (CSIR)-North East Institute of Science and Technology, Jorhat, Assam, India

## Abstract

Here, we report the draft genome sequence of crude oil-degrading *Pseudomonas aeruginosa* strain N002, isolated from a crude oil-polluted soil sample from Geleky, Assam, India. Multiple genes potentially involved in crude oil degradation were identified.

## GENOME ANNOUNCEMENT

Environmental pollution by petroleum hydrocarbons has become a global issue, with particular importance in places such as Kuwait (1–3), India (4), Libya (5), China (6, 7), and the United States (8). Crude oil contamination occurs quite often as a result of exploration, production, maintenance, transportation, storage, and accidental release, leading to significant ecological impacts (9, 10). Reducing hydrocarbons in a contaminated environment is a significant challenge. Various conventional techniques, e.g., mechanical and chemical, have been utilized in the cleanup of oil spills (11). These techniques require site restoration and are expensive. Consequently, environment-friendly techniques, like microbial degradation that will provide remediation for the degraded landmass and bring about ecorestoration, have been adapted. Many microorganisms capable of degrading crude oil have been reported to date (4, 12, 13). The majority of the crude oil bioremediation reported to date has been carried out with single or mixed bacterial strains that have the ability to grow on crude oil as their sole carbon source (14–17).

Here, we isolated the strain N002 from crude oil-contaminated soil from Geleky, Assam, India, using an enrichment culture method in M1 mineral medium with 2% crude oil as the sole carbon source. In order to isolate pure hydrocarbon-degrading bacteria, a serially diluted inoculum was spread onto solid medium and morphologically distinct colonies were purified in M1 solid agar. Further, the degradation ability of N002 was verified, and good growth was observed to occur independently in all crude oil components, i.e., aliphatic, aromatic, and asphaltene fractions. The N002 strain was confirmed to be *Pseudomonas aeruginosa* (corresponding to accession no. JX035794.1) using 16S rRNA gene PCR and sequencing.

The complete genome sequence of *P. aeruginosa* N002 was obtained by whole-genome shotgun sequencing using the Ion Torrent method. Sequencing was carried out as per the Ion 316 chip sequencing protocol provided in the Ion sequencing kit v.2.0 user guide. The genome sequence was assembled using Genome Sequencer (GS) *de novo* assembler v.2.6. The total number of reads generated using a reference-based approach was 1,074,106, with a mean length of 123 bp. The mapping of Ion Torrent 2.0 high-quality reads on the reference genome was performed using Torrent Mapping Alignment Program (TMAP) v.0.0.28 to get a consensus sequence. The open reading frames (ORFs) were predicted using Glimmer (18). tRNAscan-SE (19) and RNAmmer (20) were utilized to predict tRNAs and rRNAs, respectively. The G+C content was determined using GeneMark v.2.5 (21).

The draft genome sequence of *P. aeruginosa* N002 constituted 20.32-fold coverage, comprising 132.42 Mbp with 5,499 annotated genes and representing total genome coverage of 93.34%. The assembled genome sequence consists of 235 *de novo* assembly-based contigs with an average contig size of 2,574 bp and G+C contents ranging from 65 to 70%. The genome contains 11,038 ORFs and 5,429 protein-coding genes, 62 tRNA genes, and 12 rRNA genes. The strain N002 showed 100% homology to *P. aeruginosa* JN661695. However, the genome of strain N002 contained different hydrocarbon degradation-related genes, e.g., genes for alcohol dehydrogenase, alkane 1-monooxygenase, alkane sulfonate monooxygenase, and catecol 1,2-dioxygenase.

### Nucleotide sequence accession numbers.

The Whole Genome Shotgun project has been deposited in DDBJ/EMBL/GenBank under the accession no. ALBV00000000. The version described in this article is the first version, ALBV01000000.
